# A Review of Clothing Components in the Development of Wearable Textile Antennas: Design and Experimental Procedure

**DOI:** 10.3390/s23063289

**Published:** 2023-03-20

**Authors:** Aris Tsolis, Sofia Bakogianni, Chrysanthi Angelaki, Antonis A. Alexandridis

**Affiliations:** Institute of Informatics & Telecommunications, National Centre for Scientific Research “Demokritos”, 15341 Athens, Greece

**Keywords:** clothing components, textile technology, wearable textile antennas, wearable antenna measurements, material measurements

## Abstract

Wearable antenna systems have attracted significant research efforts during the last decade and a rich pool of review papers can be found in the literature. Each scientific work contributes to various fields of wearable technology focusing, mainly, on constructing materials, manufacturing techniques, targeting applications, and miniaturization methods. In this review paper, we examine the use of clothing components in wearable antenna technology. By the term “clothing components” (CC), dressmaking accessories/materials such as buttons, snap-on buttons, Velcro tapes, or zips are considered. In light of their utilization in the development of wearable antennas, the clothing components can play a triple role: (i) that of a clothing item, (ii) that of an antenna part or the main radiator, and (iii) that of an integration means of the antennas into clothes. One of their advantages is that they consist of conductive elements, integrated into the clothes, which can be effectively exploited as operating parts of wearable antennas. This review paper includes classification and description of the clothing components used so far in the development of wearable textile antennas with an emphasis on designs, applications and performance. Furthermore, a step-by-step design procedure for textile antennas that use clothing components as a functional part of their configuration is recorded, reviewed, and described in detail. The design procedure takes into account the detailed geometrical models required for the clothing components and the way they are embedded into the wearable antenna structure. In addition to the design procedure, aspects of experimental procedures (parameters, scenarios, and processes) that should be followed in wearable textile antennas with an emphasis on antennas that use clothing components (e.g., repeatability measurements) are presented. Finally, the potential of textile technology through the application of clothing components into wearable antennas is outlined.

## 1. Introduction

In recent years, wearable wireless devices are attracting significant technological attention within the framework of remote health care, physical training monitoring, emergent rescue services, space, and military applications. The wearable antenna constitutes a key component of wearable technology by implementing the wireless connectivity of the on-body electronic devices to the surrounding medium. The growing scientific interest in wearable antenna technology that enables compact and intelligent on-body systems has led to the development of the so-called smart garments. Smart garments are clothing items with integrated wireless sensing, localization, and communication functionalities that facilitate and support users’ personal and professional daily activities; yet the same wearability of the traditional clothing should be maintained. Considering the unobtrusive and ergonomic incorporation of on-body devices into smart clothes, the wearable antenna needs to be of compact size, flexible, and lightweight to guarantee convenient human body movement while it exhibits robust operating performance. According to the literature, there are two main categories of wearable antennas: textile and non-textile antennas. Textile antennas inherently provide flexible and lightweight solutions that can be easily integrated inside garments by partially or fully utilizing textile materials for both the conductive elements and the supporting dielectric substrates. In contrast, the non-textile antennas do not employ textile items for the fabrication of the antenna’s conductive and non-conductive parts. Therefore, this type of wearable antenna needs to be made of flexible and low-profile rigid materials to facilitate the attachment to clothes without obstructing the user’s wearing comfort. Apart from these wearable antenna categories, textile antennas that use clothing components (CC) as means of radiation and/or integration into clothes have considerably gained scientific interest. Typical clothing components which can be applied to wearable antenna technology are buttons, snap-on buttons, Velcro tapes, and zip fasteners.

In the literature, various instructive reviews can be found covering different aspects of wearable antenna technology. In [1], an overview of both textile and non-textile antennas is provided with an emphasis on issues related to materials selection and antenna design and fabrication. In [2], a detailed survey of wearable antennas for off-body radio communication in the very high-frequency (VHF) and ultrahigh-frequency (UHF) bands (below 1 GHz) is presented. In [3], recent material technology and new fabrication methods are discussed. Wearable antenna designs, miniaturization techniques, and applications are, also, examined. In [4], the authors point out various approaches to the technology of flexible wearable antenna sensors. Furthermore, in [5], the authors focus on the development of electro-textile wearable antennas with respect to materials, antenna designs, and manufacturing procedures. In [6], the authors present an extensive review of conductive textile materials and their use in flexible sensors and antennas. In [7], the authors emphasize on embroidery fabrication techniques and wearable textile antennas, in general. Further, in [8] the authors describe fabrication methods for flexible wearable antennas including textile ones. An extensive review of flexible and textile wearable antennas is presented in [9]. This review is conducted in terms of applications and focuses on the need for flexible antennas. Fabrication methods are described, as well. This review can be considered as a small encyclopedia of flexible and textile antennas for starting research in the field of flexible and textile antennas. Finally, wearable antennas from the 5G technology perspective are presented in [10]. This article presents “a perspective of the future technology landscape in wearable antennas for 5G networks”. The aforementioned articles cover different areas and subjects that engulf the wireless wearable technology.

Upon these surveys, the primary target of this article is to generate a thorough review of typical clothing components (e.g., buttons, snap-on buttons, Velcro, and zips) that can be applied to wearable textile antenna technology. The performance significance of these CCs in terms of wearability, low-profile, and reconfigurable operation use along with potential wearable antenna applications will be reported. The vital perspective is to exploit available metal structures on clothing as radiating parts of wearable antennas. Button and zipper antenna designs can be considered promising candidates in wearable textile antenna technology. Such antennas would take advantage of the electrical (e.g., conductivity) and mechanical (e.g., rigidness) properties of the metallic CCs. A similar perspective is noticed for the snap-on button and conductive Velcro components that could act both as antenna parts and clothing items at the same time. Furthermore, the CCs can contribute to the textile antenna integration inside the garments providing a stable and user-friendly environment and minimizing potential distortion of the antenna performance. The second target of this article is to record, review and describe a specific design procedure from scratch to reach a final operating prototype of a wearable textile antenna that uses CCs. The presented procedure is addressed through research studies conducted by the authors of this article and research works found in the literature. Through this procedure, challenges that are linked to the materials and geometry of the CCs are presented. Furthermore, the experimental investigation (antenna parameters, evaluation scenarios, and measurement techniques) of wearable textile antennas with emphasis on the nature of CC is reviewed and described. Finally, the potentials of the textile technology by implementing CCs within wearable antennas are described. Potentials such as the textile market expansion into antenna technology, special manufacture of CCs that are appropriate for wearable applications, low profile and user-friendly wearable textile antennas (WTA), and smart clothes designs for various applications are discussed.

The paper is structured as follows: In Section 2, CCs that operate as wearable textile antennas (WTA) or as parts of the antennas are extensively described. Regular buttons, snap-on buttons, Velcro tapes, and zip fasteners are reported in terms of geometry and performance properties. Section 3 outlines the design procedure of WTA using CCs (i.e., concept selection, textile materials, design of a reference antenna model and a CC textile antenna, and CC antenna design validation). Section 4 describes the experimental evaluation of WTA in terms of parameters, scenarios, techniques, and special experiments/measurements on the evaluation of a CC textile antenna. Section 5 presents the textiles technology potentials within wearable antennas. Finally, in Section 6 the conclusions are drawn.

## 2. Clothing Components in Wearable Textile Antennas

In this section, various categories of typical clothing components operating as autonomous radiating elements or as parts of a WTA are described. An extensive presentation, classification, and recording of such components follows.

### 2.1. Buttons

The buttons category can be classified into two subcategories: the regular buttons and the snap-on buttons. Here, we should highlight that regular button antenna designs can act as the main radiators while the snap-on buttons can possess functional and/or structural roles in textile antenna configuration.

#### 2.1.1. Buttons

Several researchers have successfully proposed button designs as wearable antennas [11,12]. In [11], a metallic G-shaped cuff button antenna is proposed for Wireless Local Area Network(WLAN)/Bluetooth wearable applications at 2.45 GHz. The antenna achieves satisfactory omnidirectional radiation patterns that are necessary for wireless data transfer with adjacent electronic devices. In [13], commercial jeans buttons have been electrically characterized for possible antenna use in industrial, scientific and medical (ISM) applications. The microstrip-fed buttons resonate at the 4.5 GHz band and present monopole-like radiation properties in terms of radiation pattern and far-field gain. The measured maximum gain is 2.2 dBi. In [14], a miniaturized button-shaped triangular patch antenna has been proposed for WLAN biomedical applications at 5.25 GHz. A frequency selective surface (FSS) has been applied as a superstrate layer to enhance antenna gain. The proposed antenna configuration shows a measured gain of 5.1 dBi over the frequency band of operation.

Furthermore, various research works have developed dual-band antennas (2.4 GHz and 5 GHz) [15,16] for on- [17] and for off-body operation [18,19,20] following almost standard jeans-shaped buttons [21]. In [15], a dual-band antenna has been developed for wearable WLAN applications. The total size is practically similar to that of a trouser button. The measured radiation patterns are omnidirectional at both operating frequencies (2.4 GHz and 5.2 GHz) to enable wireless connectivity with body-worn or elevated external devices. The gain is estimated to be close to 2.4 dBi. Similar work has been presented in [16] and in [17] presenting an upgraded antenna button design in terms of size reduction. In [18], a dual-band button antenna has been designed and experimentally validated. The proposed antenna presents a quasi-monopole radiation pattern at 2.45 GHz and a quasi-broadside radiation pattern at 5 GHz to be applied for on- and off-body wireless communication, respectively. The measured antenna gain is 1.05 dBi at 2.45 GHz and 4.5 dBi at 5.2 GHz. High radiation efficiency is, also, achieved accompanied by a small button configuration. Antenna resonance and radiation performance seems to be slightly affected by the presence of the human body due to the large ground plane. Similar work has been presented in [20]. In [19], a dual-band dual-mode E-shaped button antenna has been presented for body-centric applications. An omnidirectional pattern and a broadside pattern are achieved at 2.45 GHz and 5.8 GHz, respectively. Simulated and measured results show that the proposed design is robust under bending conditions and on-body environments. Further, it is easy to be integrated into clothes due to its flexible ground plane. In [22], a dual-band G-shaped antenna cuff button antenna has been designed. The button antenna operates at 2.45 GHz (WLAN/Bluetooth technology) and 5.8 GHz (HiperLAN technology) presenting omnidirectional radiation patterns. It should be noticed that the button shape antennas without a substrate fill [23] are less sensitive to feeding due to the lack of a dielectric layer. Frequency-tuning degradation due to structural deformation or insufficient electrical characterization of the substrate and potential humidity in the substrate is, also, avoided [16]. On the other hand, button designs using a dielectric substrate are reduced in size [24,25]. Finally, a miniaturized dual-band button antenna has been presented in [26] for wireless body area network applications. The antenna consists of a spiral inverted-F radiating part and a metal flange. In the lower band of 2.45 GHz, the antenna shows an omnidirectional pattern while in the higher band of 5.8 GHz a broadside pattern is achieved. The measured antenna peak gains on tissue phantom are −0.6 dBi and 4.3 dBi, respectively. A dual-band and dual-polarized button-shaped antennas are proposed in [27]. The antenna operates at 2.45 and 5.8 GHz. At the lower frequency band, the antenna yields linear polarization and radiates as a top-loaded monopole, being appropriate for on-body communication. At the higher band, it yields right-handed circular polarization (RHCP) and radiates as a crossed dipole, being appropriate for off-body communication. The respective gains are similar with those of a dipole (on-body) and a patch (off-body) antenna. Additionally, an innovative dual-band dual-polarized button antenna is proposed in [28]. The button is miniaturized (diameter = 19.5 mm). The antenna operates at 4.5–4.61 GHz and at 5.04–5.5 GHz Unlicensed National Information Infrastructure (U-NII) 5G Internet of Things (IoT) and WLAN. At both operating bands, vertical and horizontal linear polarizations are propagated. The final antenna is assembled to a 10 × 1 array. The antenna is fed by two ports. Both ports are of similar scattering parameters (reflection coefficient S11, S22). This makes the antenna appropriate for MIMO and for off-body communications.

Additionally, ultra-wideband (UWB) button-shaped antenna designs are proposed in [29,30,31]. The UWB technology promises high-speed data transmission at low-cost for short-range communications making it a good candidate for wireless on-body networks. In [29], a compact metallic button design has been proposed to operate in the 3.1 GHz to 10.6 GHz frequency band. The radiation patterns are omnidirectional and the achieved gain ranges from 3.45 dBi to 5.6 dBi across the operating UWB band. In [30], a button antenna design is developed by using the circular metal nail found in some buttons for size reduction. Simulated and measured results indicate that the antenna is able to operate in the UWB band. The radiation patterns show good omnidirectionality with measured gain values from 1.9 dBi to 3.4 dBi within the frequency band. The proposed button antenna can be used on a textile jacket. Additionally, an UWB state-of-the-art pattern diversity dual-polarization button antenna is proposed in [32]. Two ports are applied to this antenna. The first port yields UWB operation (4.12–9.12 GHz) with a wide 3 dB axial ratio AR (5.71–8.69 GHz) and a maximum gain of 8 dBi. The second port yields multi-resonant and multi-mode operation at 2.45, 3.7, and 5.85 GHz with modes TM01 (Omnidirectional), TM11 (Broadside), and TM21 (Side-Omnidirectional) of linear polarization. The gain ranges from 1 to 6.6 dBi which is appropriate for on- and off-body wireless links.

Button antenna designs have also been proposed for the mm-wave frequencies where multi-gigabit data transmission rates for very short-range communication scenarios are enabled [33,34,35]. In [33], a compact patch antenna in the shape of a shirt button is designed to operate at 62 GHz with a wide impedance bandwidth. The radiation pattern is directional with a maximum gain of almost 5 dBi. The antenna performance is quite stable under tissue phantom conditions. In [35], a disc-like antenna has been designed and experimentally validated for body-centric applications. The antenna operates at a 61 GHz frequency band with an omnidirectional radiation pattern of vertical polarization. The measured maximum gain is 4.7 dBi. The presence of a tissue phantom impacts the antenna radiation performance.

A magneto-dielectric button-shaped broadband patch antenna operating at 868 MHz has been reported in [36]. To decrease the antenna size for compatibility with a jacket button while maintaining its impedance matching and radiation properties, a barium-strontium hexaferrite has been employed. A low radiation efficiency of 1.5% is observed which is mainly attributed to magnetic losses.

A state-of-the-art circularly polarized button antenna is proposed to operate between 5.47–5.725 GHz U-NII band in [37]. The gain of this antenna lies between 2 and 5 dBi. The antenna presents a gap-loaded loop design of small dimensions (diameter = 2 cm). The antenna is unidirectional. A unique button-like antenna with circular polarization operating at 5 GHz for wireless body area network (WBAN) applications has been described in [38]. The antenna yields a characteristic omnidirectional pattern with circular polarization (CP) that is appropriate for on-body communication. The 3 dB AR ranges between 5.79–5.9 GHz. This is the first button antenna that combines omnidirectional radiation with CP. A wideband button antenna that operates at 2.45 GHz with 0.658 GHz impedance bandwidth and 1.8 dBi gain is presented in [39]. Bandwidth and gain are enhanced by the dielectric loading of the body whilst radiation efficiency is decreased due to losses. The wideband characteristics make the antenna robust to various environmental conditions and enable great manufacturing tolerances. Different positioning above the ground provides different radiation patterns making the antenna suitable for on- and off-body communications depending on location.

**Figure 1:**
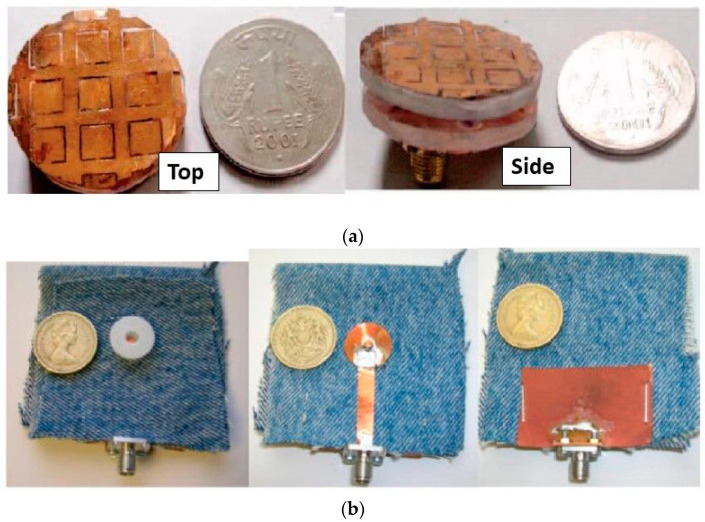
(**a**) Fabricated triangular patch button antenna: “Reprinted/adapted with permission from Ref. [14]. © 2014, © Bappaditya Mandal”; (**b**) UWB dielectric button antenna (Top-, Middle-, Bottom-layer): “Reprinted/adapted with permission from Ref. [30]. © 2007, © Benito Sanz-Izquierdo”.

Conclusively, a button-shaped antenna is inherently low-profile and user-friendly since a button is a typical part of clothes. A button topology presents a simple structure and can be easily integrated into clothing by applying ordinary sewing [15]. These features make a button-shaped antenna a suitable candidate in wearable antenna technology. Based on the recorded research works, it is obvious that the button antenna designs can cover different wireless needs within wearable technology. In Table 1, the button antennas’ structural and performance characteristics are summarized. In Figure 1, configurations of button antennas found in the literature are shown revealing the button-adjusted shape design of the antennas.

#### 2.1.2. Snap-On Buttons

Snap-on buttons are widely used CCs. Still, few researchers have studied their potential use in terms of wearable antenna design and implementation. In [40], the use of commercial snap-on buttons for achieving a modifiable textile antenna design has been presented. The aim is to accomplish resonance frequency reconfigurability. Specifically, a foldable patch antenna is designed that resonates at 8 or 9, or 10 GHz. By simply folding the textile radiator at specific lengths, the resonance frequencies can be varied. The applied design technique allows a practical and agile solution to modify the system response.

Similar work was presented in [41] where a planar antenna configuration with detachable radiating parts was developed to offer polarization and resonance frequency reconfigurability. By using snap-on buttons, as radiofrequency (RF) connectors and mechanical holders, multiple microstrip patch antenna designs can be manually interchanged. For instance, a patch antenna design that enables both right-hand circular polarization (RHCP) and left-hand circular polarization (LHCP) is obtained at 5 GHz. Further, a planar inverted-F antenna (PIFA) that resonates at 2.4 and 5.3 GHz WLAN in an alternating manner is presented. It should be highlighted that all antenna configurations are designed on a common feeding structure which consists of a two-layered substrate, five snap-on buttons, a ground plane, and a proximity-coupled feedline. Measurements of the antenna prototypes in free space, on a body tissue phantom, and in bending conditions indicate that modular interchangeable designs can provide easy, manual, and versatile reconfigurability at low cost for wearable applications. A similar research study was described in [42] where a circular patch is presented by introducing a rectangular flap in the middle of the antenna structure. This modular antenna provides interchangeable RHCP, LHCP, and linear polarization (LP). Further, resonance frequency reconfiguration in LP mode is allowed by rotating the antenna and changing the flap states (open or closed).

Additionally, the concept of employing commercial snap-on buttons as shorting pins in a textile patch antenna is presented and experimentally validated in [43]. Specifically, the patch antenna can apply different shorting pins arrangements through four sets of two back-to-back male snap-on buttons soldered into a two-layer substrate. By connecting specific male buttons in the female parts onto the ground plane, different antenna radiation patterns can be generated. Furthermore, in [44] a dual-band textile patch antenna with frequency reconfiguration has been presented. The antenna presents tuning ranges of 32.8% and 8.8% for its two frequency bands of operation at 2.45 and 5.8 GHz ISM bands, respectively. The principal idea was to embed the active components (two in-parallel varactors) and the biasing circuit into a compact and mechanically robust module of back-to-back male snap-on buttons.

In [45], a textile PIFA is presented by applying a coplanar frequency reconfiguration mechanism. The reconfiguration module includes a compact printed circuit board that embeds the active tuning circuit with snap-on buttons offering the electronic-to-textile connection. The antenna prototype achieves a wide operating frequency range of about 70%. Finally, in [46] a coupled-mode substrate-integrated cavity (CMSIC) antenna is presented. The antenna achieves radiation pattern reconfiguration at 2.45 GHz by employing two snap-on buttons as passive switches. When the buttons are in the OFF state, the antenna generates a broadside radiation pattern with a maximum gain of 9.2 dBi, while, in the ON buttons state, the radiation pattern is omnidirectional with a maximum gain of 3.1 dBi.

**Figure 2:**
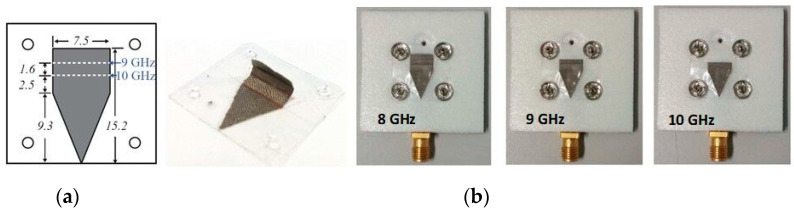
(**a**) Foldable patch module (dimensions in mm). The white dash lines indicate the radiation element folding positions for the 9 and 10 GHz configurations; (**b**) prototype module realizations. “Reprinted/adapted with permission from Ref. [40]. © 2016, © Shengjian Jammy Chen”.

In Table 2, the snap-on button antenna geometry and performance properties are recorded. In Figure 2, a topology of an X-band WTA using snap-on buttons for reconfiguration purposes is shown revealing the flexibility offered by the use of such kind of CC.

### 2.2. Velcro

A limited number of research works that apply the Velcro CC as an active part of WTAs have been recorded in the literature. A lot of researchers though have used Velcro tapes as a means of integrating wearable antennas into the garment. This differentiation can arise from the fact that non-conductive and conductive Velcro tapes can be found in the textile market. The non-conductive tape can be mainly used for antenna integration. Still, it can be used as part of the antenna to facilitate interconnection with a textile feed line, as shown in [47]. Specifically, in this work, the use of a plastic Velcro enables an all-textile connector for a textile patch antenna. On the other hand, the conductive Velcro can be successfully used as a functional part of wearable textile antennas, as proposed in [48]. The use of Velcro tapes provides polarization and frequency reconfigurability into the patch antenna. The Velcro-base (hooks) has been sewn with a non-conductive yarn onto the patch layer providing mechanical stability. It should be noted that slight degradation in the antenna radiation efficiency has been noticed due to the ohmic losses of the conductive Velcro. Furthermore, the conducted repeatability measurements proved a stable antenna performance. Based on the research works, it seems that the Velcro as a CC used in WTA is quite user-friendly, adequately satisfying the wearability requirements.

**Figure 3:**
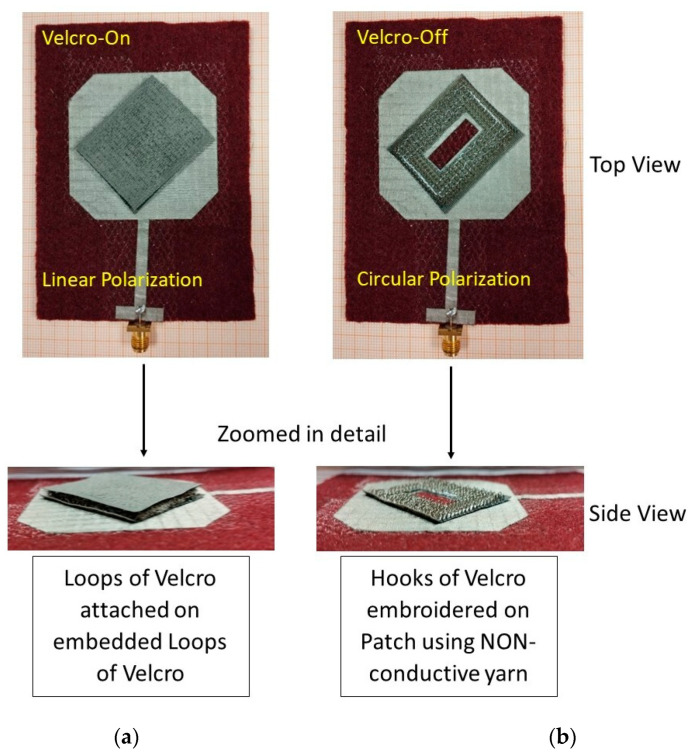
Textile reconfigurable patch antenna applying different Velcro states: (**a**) on-; (**b**) off-state, respectively.

In Table 3, details of the Velcro-based patch antenna designs are presented. In Figure 3, a photo of the textile patch antenna described in [48] is shown revealing the great potential and easiness of Velcro use for antenna structural reconfiguration.

### 2.3. Zip

The zip as a clothing component is widely used in jackets and pants. Still, few researchers have examined its potential use in wearable antenna designs. A zip antenna can present a lot of advantages such as low cost, compact size, planar flexible structure, and simple garment integration. Owing to these features, a textile monopole antenna based on a commercial zip fastener is proposed for 2.5 GHz WLAN applications in [49,50]. The zip antenna achieves an omnidirectional pattern with a realized gain of about 0 dBi. The antenna tunable capability is examined. Furthermore, a handbag metal zipper is presented in [51] to act as an off-body antenna. It is found that the zip antenna can operate at a satisfactory level for both “user cases”, closed and open. The textile antenna can present multiple resonant frequencies by shifting the feeding point and can generate different radiation directivities by opening or closing the handle of the zipper. At 2.44 GHz, the zip antenna gain is almost 5 dBi. Similar to the handbag zipper, a jacket zipper antenna is presented in [52] demonstrating the natural radiation pattern reconfigurable capabilities of a zip antenna. The antenna operates at various frequency bands depending on the feeding position (1.5 GHz and 7.5 GHz or 2.5 GHz). The zipper can efficiently radiate in omnidirectional, multi-lobe, and omni-like modes when the zip is fully closed, half-opened, and opened, respectively. The achieved gain ranges from 0.9 to 8.9 dBi at various frequencies. This work also validates the tunable reconfigurable nature of a zipper, proving the great potential and capabilities of such CC as the main radiator.

Furthermore, in [48], a textile patch antenna that includes a fully conductive zip (teeth and cloth holding the zip) has been proposed by the authors of this review paper. The zip is embedded into the patch layer to provide polarization and frequency reconfigurability avoiding the use of complex active circuit elements. The zip serves as a polarization actuator. The work has been partly presented in Section 2.2. It should be highlighted that the conductive zip can shift the antenna resonance frequency downwards due to the elongation of the current path on its structure. Furthermore, due to the ohmic losses of the conductive zip, degradation of the antenna radiation efficiency has been noticed. Still, the repeatability measurements showed acceptable performance and stability of the zip patch antenna.

**Figure 4:**
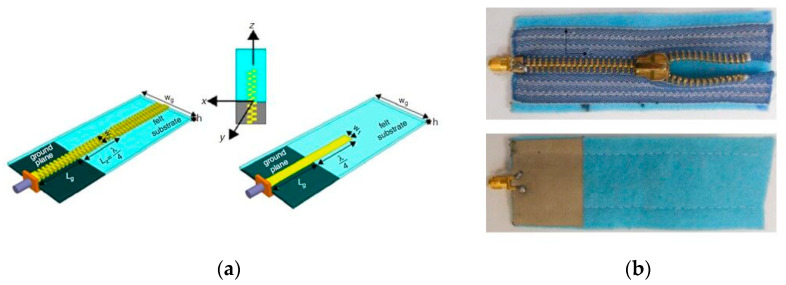
Zip monopole antenna: (**a**) models; (**b**) prototype: “Reprinted/adapted with permission from Ref. [49]. © 2011, © Mohamad Mantash”.

Conclusively, the main advantage of the zipper is the variety of different open state positions which could be electromagnetically used to offer reconfigurability in the antenna design; still, the challenging part is its electromagnetic modeling complexity. Further, a zip-based antenna seems to be user-friendly. In Table 4, details of the zip antennas are recorded. In Figure 4 an example of a zip monopole antenna (model and prototype) is shown revealing its simplicity and ease of use.

### 2.4. Discussion on Research Data of Wearable Textile Antennas Characteristics and Performance

As can be seen from Table 1, Table 2, Table 3 and Table 4, a considerable number of wearable textile antennas that use clothing components have been developed to enable wireless body-area applications. Various wireless standards such as WLAN, Wi-Fi, ISM, Bluetooth, UWB, WiGig, and X-band can support wearable applications. The proposed button antenna designs with monopole-like behavior present satisfactory radiation characteristics similar to a monopole antenna. Further, the button antennas with patch-like behavior can mostly achieve high radiation efficiencies. Adequate resonance and radiation performance are, also, provided by the snap-on antenna designs. It should be noted that the dimensions of the buttons and snap-on buttons WTA are within or lower than the expected conventional size (or equivalently electrical size) with a maximum length of 6 cm. The Velcro WTAs present sufficient performance properties with slightly increased dimensions compared with the button patch-like designs at similar operation frequencies (maximum dimension of 10 cm). The zip antennas present good monopole-like characteristics with a compact (5.7 cm) or increased length (38.5 cm) in the case of the handbag zipper. The impedance bandwidths obtained by the CC textile antennas can cover from narrow bands such as 90 MHz to increased frequency bands such as 300 MHz and 1000 MHz for increased data rates and 4–12 GHz for UWB and mm-Wave high data rates. All recorded bandwidths reveal the considerable use for 5G mm-wave and 5G sub-6 GHz communications. The achieved far-field gain values are compatible with the antenna type and the respective way of radiation. Still, it is observed that the gain is decreased compared to conventional (without CC) WTAs. This may occur due to inherent losses of the CCs (e.g., shape and materials). Generally, it has been shown that various antenna performance requirements can be covered by a CC WTA.

## 3. Design Procedure

The design of a wearable textile antenna that utilizes clothing components can be quite challenging and, to some extent, more complex compared to the design of a conventional textile antenna. The design challenges are mainly linked to the material properties of the applied CC, the detailed and complex CC geometry that needs to be taken into consideration, and the concept of applying a CC.

In this section, an effective step-by-step design procedure for textile CC antennas is recorded, reviewed, and analytically described. The recorded five steps are derived through research works presented in the literature and conducted by the authors of this paper. The five steps of the design procedure are listed below:

(a) Concept selection
(b) Materials definition and characterization
(c) Reference antenna design
(d) CC antenna initial design and modeling
(e) Final CC antenna validation

The main target of this analysis is to give an insight into the design procedure, specific challenges from an engineering point of view, and difficulties with the processes followed in each step. Emphasis is given to the special nature and design prediction of CC when employed in or as WTA. Each design step is described through examples, simulations, and measurements.

    (a) Concept Selection

**Figure 5:**
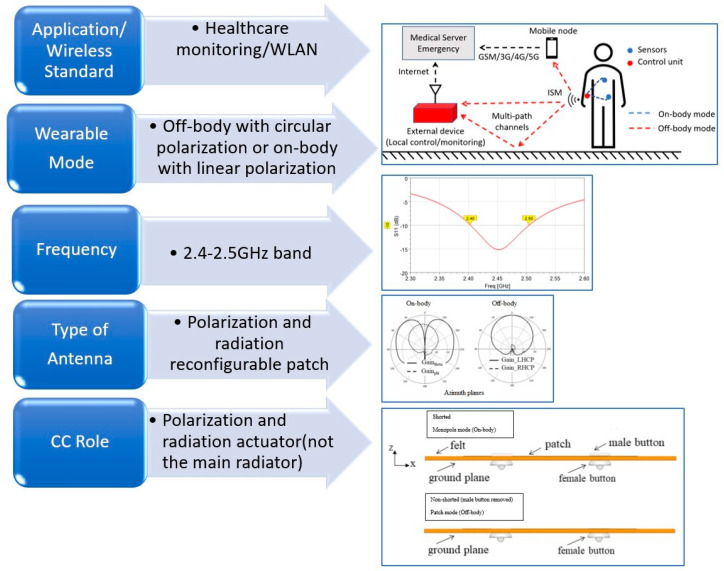
Diagram of the “Concept Selection” design step.

The primary fundamental step in the design procedure of a CC textile antenna is the concept selection. This design step deals with the determination of the antenna application type and the involved requirements and specifications. Regarding the application type, a CC textile antenna can provide the wireless connectivity within the framework of healthcare monitoring, specialized occupations (e.g., firefighters and rescue services) surveillance, or sports and fitness recording, for instance. Subsequently, the wireless communication technology that can support the desired application needs to be designated. Wireless Personal Area Networks (WPAN) (e.g., Bluetooth), WLAN (e.g., Wi-Fi), or cellular (e.g., GSM, 5G) wireless standards can be implemented to establish the communication link. Furthermore, the on-body environment in which wearable antennas operate imposes requirements that should be included in the antenna’s design criteria. The wearable antenna should be lightweight, low profile, and, present compactness and flexibility provided by the textile materials and clothing components to be specified. In this design step, the antenna frequency of operation can be assigned (e.g., 1.9 GHz, 2.4 GHz, 5 GHz) based on the selected wireless communication standard. Additionally, the wearable operation mode should be clarified to enable wireless on- and off-body communication links. All these considerations can lead to defining the type of antenna to be designed (e.g., a monopole antenna, microstrip antenna, or planar inverted F-antenna). For example, if an on-body wearable operation mode is required with an operating frequency of 2.4 GHz, then, a vertical top-loaded monopole could be a suitable candidate and could be designed by using a button configuration, as presented in Section 2. Further, in this step, and depending on the above considerations, we should determine if the CC can serve as the main radiator or if it will be part of the antenna. This first step will result in a basic scribble of our antenna to be designed. To establish this design step, a realized published example [53] is visualized in Figure 5.

To summarize step (a), the concept selection is described in short as follows: (i) application, (ii) wireless technology, (iii) frequency of operation, (iv) operation mode (on-/off-body link), (v) type of antenna, and (vi) usage of CC (as part of the antenna/or as the main radiator).

    (b) Materials definition and characterization

Generally, fabric materials and components that are widely used and easily accessed are potential candidates to apply within the textile antenna design. Still, the material selection is crucial regarding the textile antenna performance [7]. For instance, the efficiency of a microstrip antenna can be influenced by the permittivity, dielectric losses, and thickness of the dielectric substrate. In textile antenna designs, typical non-conductive dielectric cloths can be employed as substrates. Textile fabrics such as felt, fleece, denim, and cotton can be used as antenna substrates to ensure both satisfactory antenna performance and wearability. In terms of conductive materials, the textile antennas can utilize thin and uniform metallization (e.g., copper tape), woven or knitted conductive textiles, also known as electrotextiles (e.g., Nora Dell), and conductive yarns (e.g., Amberstrand) that can be sewn or be embroidered to form the required antenna pattern. The selection of the conductive textiles for the conductive antenna elements (e.g., patch and ground plane) is significant for the antenna radiation response, as well. In addition to these materials, the CC textile antennas make use of clothing components which in most cases should be conductive (Figure 1, Figure 2, Figure 3 and Figure 4). For example, a top-loaded monopole can be applied through a button design. This will require the use of a conductive button. The textile materials, prior to implementation, need to be defined and characterized in terms of their electrical properties. The determination and characterization of the materials can be specified as follows:

    *(b1*)  *Conductive cloth or yarn*

**Figure 6:**
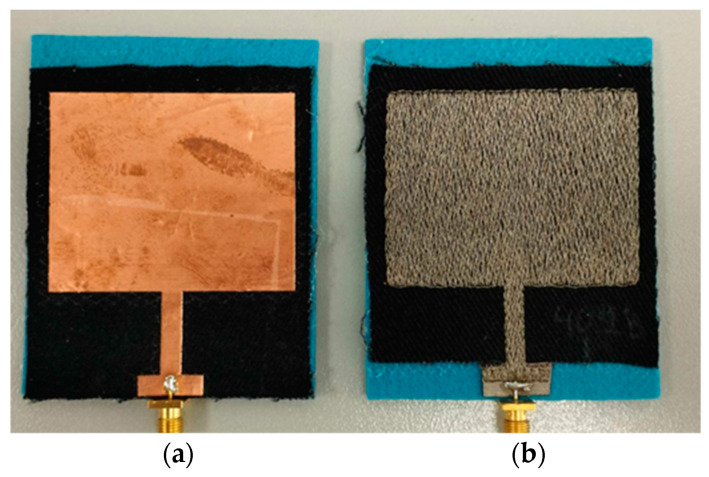
Microstrip patch antennas applying different conductive materials: (**a**) copper tape (σ = 5.8 × 10^7^ S/m); (**b**) conductive embroidered yarn (σ = 1.1 × 10^4^ S/m).

The electrical properties of the conductive materials need to be specified. In general, conductive textiles should present low electrical surface resistance to reduce the electrical losses and, therefore, improve antenna efficiency. Regarding the antenna design, the critical parameter to determine is the conductivity of the materials [6]. In the case of thin uniform metallization (e.g., copper tape), the conductivity is directly provided by the suppliers and it is on the order of magnitude 10^7^ S/m. When woven conductive cloth (e.g., Nora Dell) is used, surface resistivity is usually given by the manufacturers. The conductivity can be calculated from the resistance data and thickness of the fabric and it is on the order of magnitude 10^6^ S/m. In the case of an embroidered conductive pattern, an equivalent conductivity should be derived, as analytically described in [54]. A descent achieved conductivity of an embroidered pattern is on the order of magnitude 10^4^–10^6^ S/m depending on stitch density. Figure 6 presents a microstrip patch antenna applying conductive materials of different equivalent conductivity [55].

    *(b2)*  *Dielectric substrate material*

**Figure 7:**
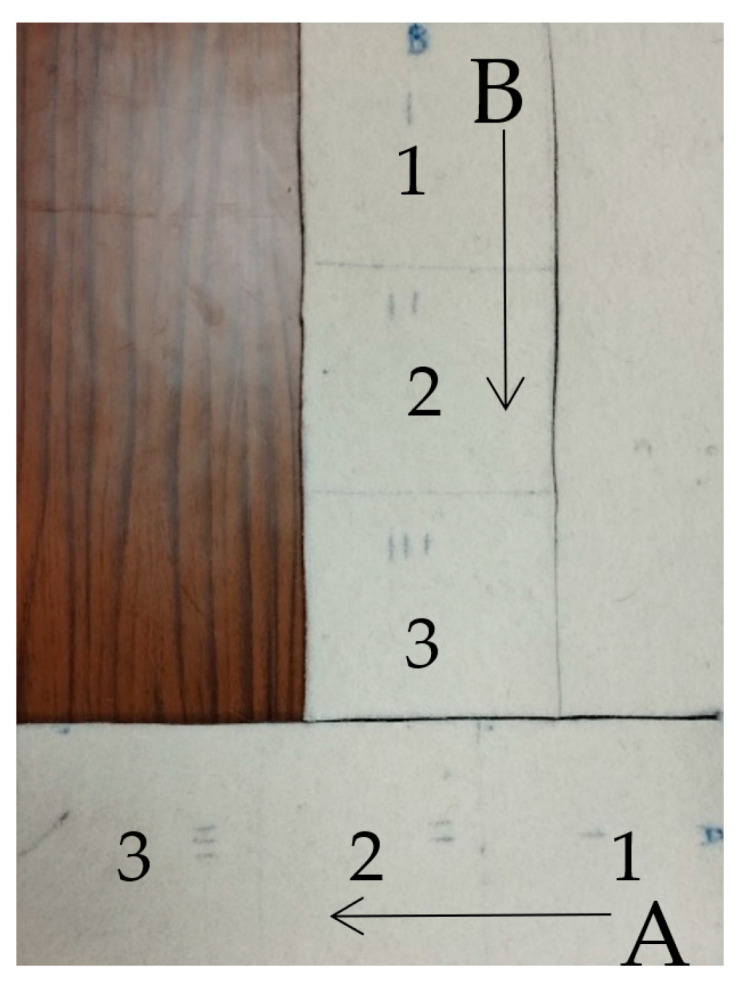
Felt material cut into different directions to be tested (A, B: different directions of felt cuts, and 1, 2, 3: different samples of each direction).

**Figure 8:**
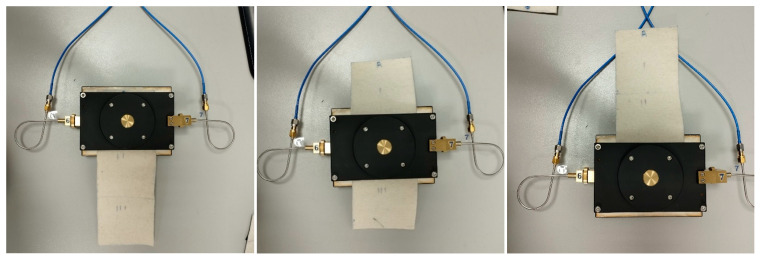
Q-meter felt dielectric measurements of three different samples cut along direction A.

Commercially available fabric materials such as felt, denim, and cotton are commonly used in wearable textile antennas. The dielectric properties of these materials are not usually known, therefore, they need to be measured since their electric behavior significantly affects antenna performance. The dielectric constant, ε_r_, and loss tangent, tanδ, are the critical parameters to be determined. Various experimental techniques can be used for the measurement of the dielectric properties including Split Post Dielectric Resonator (SPDR), Nicholson-Ross-Weir (NRW), Microstrip stub line, and other methods, as analytically described in [56]. Even if the dielectric values are provided by the suppliers, the textile parts that will be used for antenna fabrication still need to be measured since such materials are usually not homogeneous and their properties can spatially vary. In order to prove this statement a commercial white felt is cut into two orthogonal pieces along different directions A and B as shown in Figure 7. The aim is to measure the dielectric properties of three different samples in each direction at 2.47 GHz. An SPDR [57] equipped with a Q-meter is used for the measurements, as shown in Figure 8. The measured dielectric properties results are recorded in Table 5. As can be seen, direction A yields greater variation in terms of the dielectric properties e_r_ and tanδ compared to direction B. Felt seems to be more stable along direction B.

Conclusively, regarding the effective antenna design process, the textile sample that will be used for the antenna fabrication needs to be properly characterized. In the case of using multiple dielectric parts, we suggest using textile parts along the direction which yields more stable properties.

    *(b3)*  *Clothing component materials*

**Figure 9:**
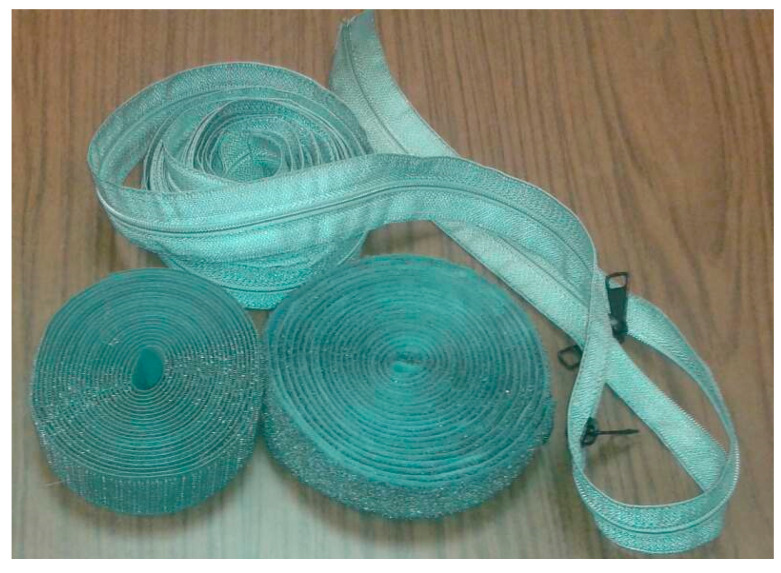
Conductive Velcro tape and Zip fastener from Shieldex, Bremen, Germany (www.shieldex.de, accessed on 2 March 2023).

Commercial clothing components such as Velcro tapes, zip fasteners, buttons, and snap-on buttons can be made of conductive and non-conductive materials. Regarding the conductive Velcro and zip accessories, their electric properties are usually given in terms of surface resistivity and their respective conductivity can be calculated. In Figure 9, Velcro tapes metalized with 99.9% pure silver and fully silver-plated zippers are presented. Due to their electrical conductivity, novel application capabilities can occur in smart textiles. The buttons and snap-on buttons are made of standard commercial metals (e.g., copper and brass) and their conductivity can be found in the literature. In the case of non-conductive CCs, the buttons and snap-on buttons are usually made of known polyester with specific properties. Furthermore, commercial Velcro tapes are made of polyamide or polyester. The non-conductive zips are made of polyester or nylon. The non-conductive CCs are usually homogeneous and bulk; still, they maintain stable properties. All non-conductive CCs properties need to be estimated and calculated as in (b2).

    (c) Reference antenna design

Reference antenna design model is a quite critical step throughout the design procedure. The targets of this step are: (i) the creation of an antenna design model as a reference one without the use of clothing components, (ii) evaluation of the properties of the textile materials, (iii) comprehension of the proposed antenna concept, and (iv) determination of the effect of realistic factors such as the SMA connector effect. The tasks that can be performed in this step are: design and simulation of the reference antenna/s (REF) to conduct the desired operation and fabrication of an antenna prototype to evaluate the numerical estimation, the properties of the applied textile materials, the realistic factors and the principle of operation of the specific antenna.

**Figure 10:**
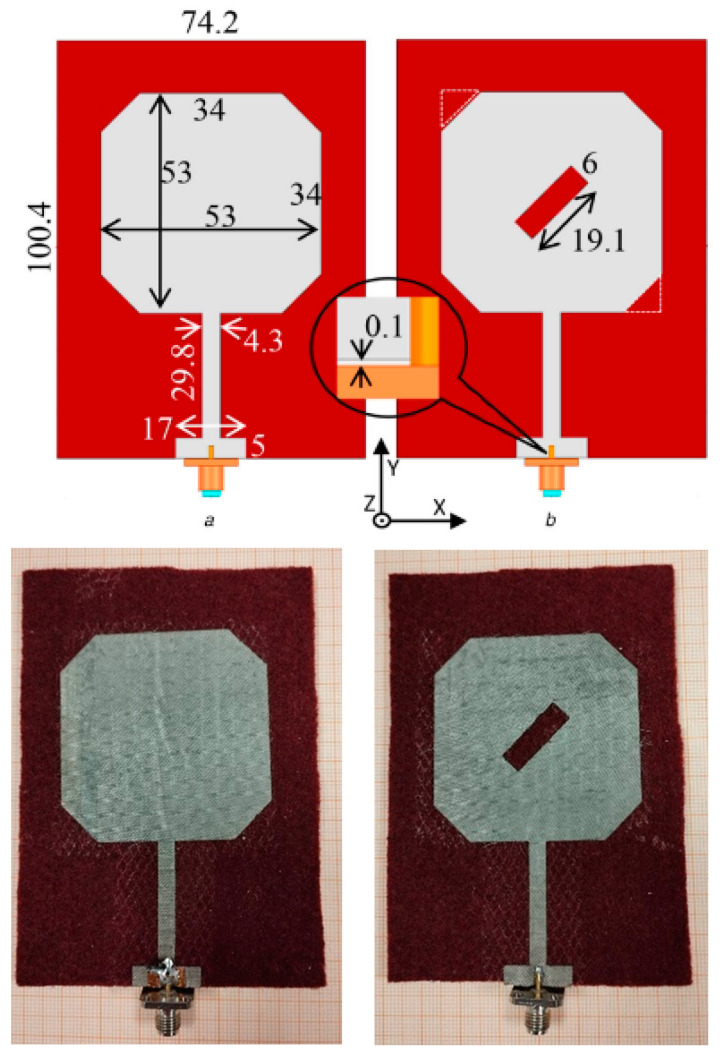
Models (**top**) and prototypes (**bottom**) of REF1 (**left**) and REF2 (**right**).

To unravel this design step, two reference antenna design models and their fabricated prototypes, which have been presented in [48], are employed. The antenna concept was to develop a textile microstrip patch antenna that presents polarization reconfiguration by applying a CC as a passive reconfiguration means for off-body wireless applications. Felt material is used for the dielectric substrate, while, the patch and ground layers are made of conductive electrotextile (Nora-Dell). The first antenna model (REF1) reproduces linear polarization while the second one (REF2) reproduces the circular polarization. The circular polarization is obtained through the introduction of the diagonal slot into the patch layer. Both principles of operation are achieved via a single antenna configuration when a CC is added. The models and prototypes of the reference antennas are shown in Figure 10.

The simulated and measured performance characteristics of both antennas are shown in Table 6. Regarding the REF1 antenna, the observed difference in the resonant frequency is mainly attributed to the fact that the antenna prototype length is found to be shorter (by 0.5 mm) than the numerical one. A slight increase in impedance bandwidth may reveal increased textile substrate losses of the prototype antenna compared to the simulated model. Furthermore, the axial ratio at the operating frequency seems to degrade, mainly, due to measurement imperfections and antenna positioner effects that are not counted in simulations. Still, the axial ratio of the prototype is acceptable and yields a clear LP. Furthermore, the measured far-field gain is decreased which can indicate two matters: the prototype presents extra losses than simulations, and the measurements unavoidably cause errors due to various factors that will be described in Section 4. Still, the gain value is suitable for off-body communication. Further, the numerical and measured radiation efficiencies are quite close. Similar observations can be deduced for the REF2 antenna. It is apparent that the measured axial ratio is at the limit of 3 dB. This occurs due to slight differences in the fabricated dimensions of the diagonal patch slot. Overall, both reference antennas yield satisfactory performance results without considerable deviations between simulations and measurements.

This design step allows us to figure out design and fabrication issues prior to the implementation of a CC that may cause perturbations in the antenna structure. Actions that can be realized to overcome potential issues are: re-measurement of dielectric substrate losses taking into account possible use of an adhesive material, re-fabrication of prototypes with more accurate techniques to achieve precise antenna dimensions, and elimination of measurement errors. It should be outlined that as long as the textile antenna requirements are met despite potential discrepancies between simulations and measurements, the re-adjustment of the reference antenna model is optional.

    (d) CC antenna initial design and modeling

In this step, the clothing component that has been selected in the concept selection step is taken into account within the design process evolving the reference antenna. This means that the antenna model is designed by integrating the CC into its structure. To accurately simulate the CC textile antenna and evaluate its performance properties, the CC needs to be geometrically modeled in detail. CC antenna performance may differ from the REF antenna’s resonance and radiation behavior. On that occasion, the CC antenna design needs to be re-adjusted to achieve the requirements settled in step (a) and fulfilled by the REF model.

**Figure 11:**
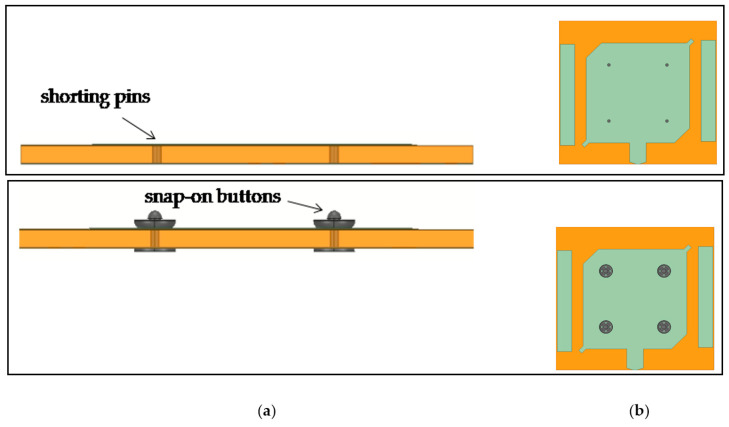
Patch antenna modeled with shorting pins (**top**) and with snap-on buttons (**bottom**): (**a**) Side views; (**b**) Top views (not on the same scale).

In Figure 11, the side view of a REF textile patch antenna that uses shorting pins and the side view of a CC antenna that applies snap-on buttons instead of pins are depicted. In Table 7, a performance comparison between the two antenna models is recorded. Based on the simulated results, minor deviations are observed between the antenna models. Therefore, there is no need for design re-adjustment of the CC antenna. Here, it should be highlighted that the design of the initial antenna model using shorting pins in place of the geometrically complex snap-on buttons considerably saves valuable computational time until the desired application requirements are accomplished. Then, the CCs can be used by applying a light design optimization scheme.

    (e) Final CC antenna validation

The final step in the design procedure is to fabricate and evaluate the CC textile antenna in terms of its expected/simulated performance. The challenging task is to diligently incorporate the selected clothing components into the antenna structure. For example, in [48], the Velcro hooks-part and the zip are sewn on the microstrip patch layer with a non-conductive yarn. This provides the necessary mechanical stability for the clothing components on the antenna. Attention needs to be given to avoid a major local compression of the textile substrate.

After the CC antenna is fabricated, performance evaluation is conducted via measurements of the reflection frequency response (S11) and the far-field radiation properties (radiation pattern, gain, radiation efficiency, and axial ratio). The fabricated antenna’s resonance response may differ from the simulated one. In this case, we should examine the prototype in terms of its geometrical characteristics that may be changed due to fabrication processes. For instance, the height of the textile substrate can be locally shrunk due to compression caused by the clothing components sewing. Furthermore, improper alignment of CC position into the antenna structure can impact the resonance response. Various research works present analytical comparison studies between simulation and measurements of CC textile antennas and factors of differences and improvements are described. In [44], the deviation in far-field gain is found to be 1 dB which is justified by imperfections in antenna fabrication and inaccuracies in measurements. Furthermore, this work proves that since the CC is modeled in detail and as accurately as possible, then, the measurements will approach the numerical results.

## 4. Experimental Evaluation

As long as the final design of the required WTA model is obtained (as described in Section 3), the textile antenna prototype needs to be experimentally characterized. The fundamental antenna parameters that should be measured are those described in the design procedure at step (e). This section will focus on the description of the WTA evaluation scenarios that need to be taken into account due to operation in a “wearable environment“. Furthermore, the measurement techniques applied to wearable antennas will be presented noting the advantages and disadvantages. Finally, this section will emphasize on the CCs experimental evaluation.

### 4.1. Wearable Textile Antenna Evaluation Scenarios

The evaluation of a WTA is a more demanding and challenging task compared to the respective one of a conventional free-space antenna in terms of the measurements and experimental scenarios that need to be realized. Two general scenarios should be examined; when a WTA operates in free space and when the antenna is mounted onto a human body. Initially, it is useful to measure the WTA performance (gain, S11, e_rad_) in free space [58,59,60]. The free space measurements can be considered an ideal case as the effect of the lossy, complex, and inhomogeneous human body environment is not considered yet. Therefore, the free-space WTA performance is an optimum one. However, the human body can strongly impact the antenna performance due to its electromagnetic, structural, and environmental effects including absorption, depolarization, creeping waves, bending, crumpling, and wet conditions [61]. A WTA will mainly operate under the movement of a human body, and specific deformations on the antenna geometry are unavoidable. Therefore, structural conditions such as bending (e.g., antenna integration on a human arm) [62,63,64,65,66] and crumpling [67,68] need to be experimentally evaluated. The WTA should be, also, measured under different environmental conditions such as high humidity to represent perspiration or exposure to water [69,70]. Additionally, the WTA is considered to be part of the garment. Testing the durability of the textile antenna in terms of performance after washing and drying is thus required [71]. The effect of the surrounding or covering cloths should be examined, as well [72,73]. Repeatability measurements in terms of the textile antenna performance should also be examined [74]. Furthermore, measurements on a real human body [75,76] or on a human body phantom [77,78] should be carried out. These antennas should be measured in terms of S11, near-field (if possible), and far-field parameters [79,80,81,82,83]. Furthermore, the Specific Absorption Rate (SAR) should be evaluated as well in terms of real applications and safety requirements.

### 4.2. Measurements Techniques and Methods

Antenna measurements can be conducted in an anechoic chamber or in a real environment (outdoors [71], and indoors). As previously noted, important parameters that need to be extracted from measurements are reflection coefficient S11, E-field distribution in the near field region, and far-field parameters (radiation efficiency, gain, directivity, and radiation patterns).

Prior to the description of WTA measurement techniques, it should be mentioned that simulation models by employing numerical phantoms need to be used depending on the application (e.g., arm, head, whole body, homogenous, layered, and flat phantoms) [61,81,84,85,86,87,88,89,90,91]. On-body antenna simulations are of great importance since they save time in terms of optimizing the antenna performance before proceeding to antenna measurements on a body/phantom. The design procedure presented in Section 3, steps (c) to (e), should be repeated by using a numerical phantom since the first run is for free space operation. Then, the antenna design parameters are re-adjusted to obtain the final optimum CC WTA design and prototype. Alternatively, the proposed design process can be realized by directly employing a numerical phantom in simulations from the beginning of the design process. It should be noted that potential differences from free space can be accepted as long as the operational requirements are at an acceptable range.

The critical question for all measurement methodologies that have been applied so far is: what is their contribution to the evaluation of WTA performance, and what is the influence of the body/phantom? It is useful to address one by one all WTA performance parameters and highlight for each one the difficulties, challenges, and advantages that exist to evaluate them.

The reflection coefficient (S11) of a WTA expresses the input antenna impedance (Zin), which is highly affected by the presence of the human body. Therefore, it is critical that the S11 parameter should be measured on-body to assess the antenna resonance response under this lossy surrounding medium. A lot of papers have successfully presented on-body/-phantom S11 measurements in various locations and under various postures [92,93,94,95,96,97,98]. Furthermore, it is important to evaluate the repeatability of the S11 results by realizing measurements on different bodies and phantoms, if possible. This will provide an average estimation of the S11 and will allow a more accurate evaluation of antennas which are highly influenced in terms of Zin by the presence of the body/phantom. Such antennas are dipoles or monopoles where there is no ground plane to shield the radiation from the human body [99].

The radiation efficiency, gain, directivity, and radiation patterns are fundamental antenna parameters that can be directly measured in a far-field test site. However, measurement setups with phantoms introduce difficulties in such test sites. Specific equipment (positioner) is required to rotate the phantom in the elevation and azimuth axis to obtain the 3D radiation patterns. If the phantom is liquid or semi-solid (gel) [73], there is a risk of breaking or spilling the liquid of the phantom while rotating (scanning) it on the roll/elevation axis. Therefore, manual rotation is required to effectively capture multiple elevation angles. In case the phantom is solid, the rotation of the phantom on the roll axis may be more effortless. Still, there are two main concerns for the solid phantom: (i) it is quite expensive compared to other types of phantoms and (ii) it is heavy so a firm-duty positioner is required [82]. This concludes to the fact that in far-field test sites, the easiest and most feasible solution is to solely rotate and scan the phantom on the azimuth plane [77,78,100,101,102]. This leads to measuring far-field antenna parameters only on the 2D plane around the phantom [103]. The above restriction is mostly valid in the case of the torso, or whole-body phantoms [82]. In the case of applying an arm or head phantom, it is easier to rotate the phantom on the roll/elevation axis to acquire a second plane and, finally, obtain the desired 3D radiation pattern.

Regarding the radiation efficiency, it is proven that it can be measured with satisfactory accuracy in a reverberation chamber (RC), while the antenna is mounted onto the body [76,104,105,106,107,108]. Therefore, the reverberation chamber is an efficient, though expensive, solution for wearable antenna efficiency measurements. This solves the issue of producing 3D radiation patterns to calculate the efficiency on a body/phantom. Still, this measurement technique lacks in providing images of the E-field distribution in the near field region, gain, directivity, and the radiation patterns of wearable antenna. The wearable antenna radiation efficiency has, also, been successfully measured by a modified Wheeler cap [83].

**Figure 12:**
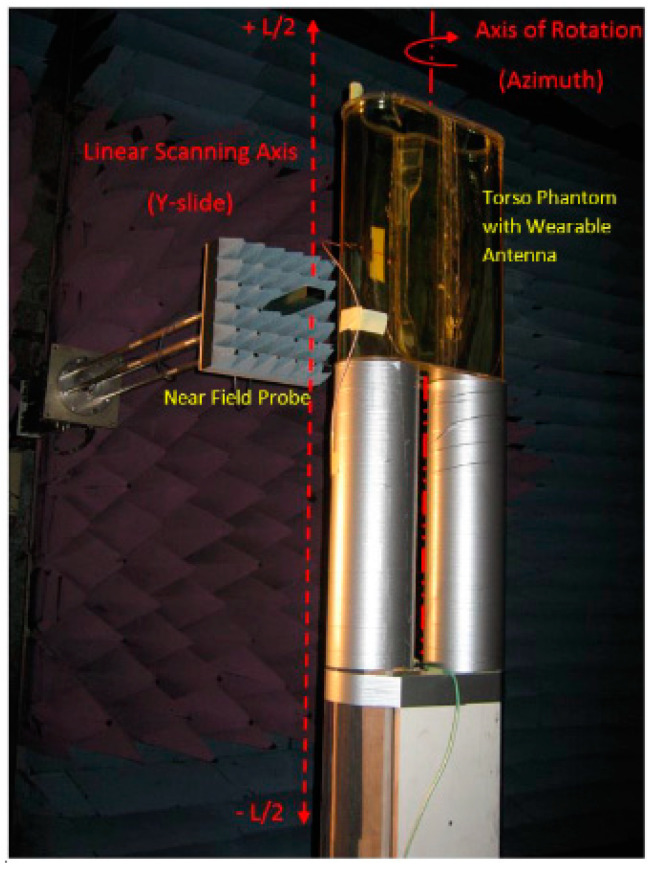
Cylindrical Near Field measurement setup.

All antenna parameters described above can be measured by using near-field measurements (spherical and cylindrical). In the last couple of decades, the spherical near-field measurement technique (STARGATE SG-62 [109] and RAMS [110]) has been successfully used as an alternative method for assessing the far-field radiation of antennas in close proximity to the human body (handset antennas). This indicates the growing interest in using the near-field measurement technique for wearable antenna performance evaluation [111,112,113]. Furthermore, the cylindrical near field technique (CNF) (Figure 12) has been successfully proposed for effective WTA antenna measurements [114]. CNF is considered to be one of the best and most competing techniques in terms of measuring antenna parameters and facilitation of measurements. The advantages of this technique can be listed as follows: less physical space is needed compared to the far field measurement; the phantom needs to be mounted on a turntable which rotates only on the azimuth axis, excluding rotation on the roll axis (as it will be conducted when using the Spherical Near Field measurement technique); information about the image of the E-field distribution in the near field region of the phantom by the antenna is acquired by the near field scan which could be important for the best position selection of the wearable antenna leading to a better design of a wearable antenna system and far-field results (gain, directivity, efficiency, and radiation patterns) can be produced by transforming the near field ones. The measurement potential of the CNF technique for evaluating wearable antennas is that it can capture several possible antenna positions with a single scan due to the approximate cylindrical shape of the body/phantom (the CNF measurement technique lacks in capturing sampling data at the top and bottom of the AUTs elevation plane). This can reduce the measurement time and the required volume of the test site.

Another important parameter that should be evaluated is the specific absorption rate (SAR) so that the exposure of the human body to the EM-fields produced by the WTA is kept at limited levels defined by ICNIRP and IEEE standards [115]. The assessment of SAR is critical for safety reasons and constitutes a sufficient electromagnetic dosimetry measure for wearable applications. International guidelines determine the maximum permitted values of the SAR to guarantee the user’s safety. For example, the IEEE C95.1-1999 international standard limits the SAR averaged over 1 g of tissue in the shape of a cube (SAR-1g) to values lower than 1.6 W/kg for general public exposure [116]. Furthermore, the IEEE C95.1-2005 standard limits the SAR averaged over 10 g of tissue (SAR-10g) to values lower than 2 W/kg [117]. Therefore, compliance with international guidelines needs to be examined.

The above-described measurements follow the principle of evaluating the effect of the lossy body/phantom on antenna performance. SAR follows the crucial evaluation of the impact of the antenna operation on the body. This is the reason for reporting this separately. The estimation of SAR can be realized via simulations and measurements [84]. The calculation of SAR should be taken into account during the design procedure to define potential changes in antenna geometry (e.g., an extension of the ground plane [118,119,120]), adjust the position and distance of WTA [121] with respect to the body, and constrain the power inserted into antenna input if required to be reduced (with possible cost in the link budget). Power reduction by ground plane extension has been experimentally examined in [122]. Furthermore, controlling the SAR by using an artificial magnetic conductor (AMC) structure in the textile antenna has been presented in [123]. The shielding structure which is slightly larger than the antenna reduced SAR by 90% maintaining the flexibility of the antenna.

A lot of research works have successfully presented simulations and measurements of SAR. In [14,18,20,46,124] simulation models for calculating SAR values are presented. Specifically, in wearable antenna design simulations, a numerical tissue model needs to be applied to evaluate SAR results. Maximum SAR values can be estimated for a specific antenna input power at the frequency of operation. SAR distributions in the surrounding tissues can, also, be generated. In [26] and in [125] measurements of SAR regarding a button antenna and a jacket-like antenna are provided, as well. SAR can be measured by two experimental techniques [126]. The first technique is the electric field probe method and the second one is the thermographic method. The first one is time-consuming and expensive, but with low complexity. On the other hand, the thermographic method is cheaper; still, it requires more components and is not suitable for real RF devices. In general, the measurement setup can include the textile antenna, the tissue phantom, and the SAR measurement probe or the thermographic camera as basic parts.

### 4.3. Clothing Component Experimental Evaluation

**Figure 13:**
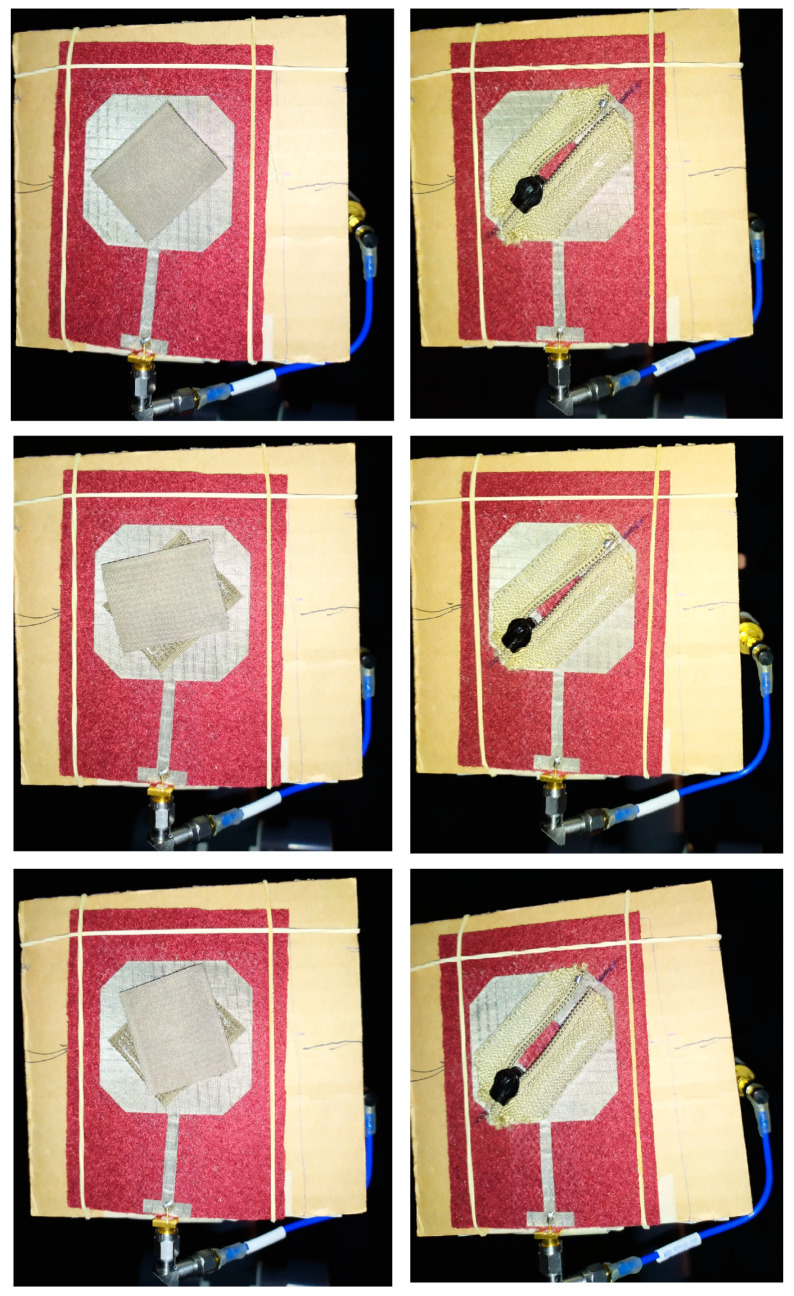

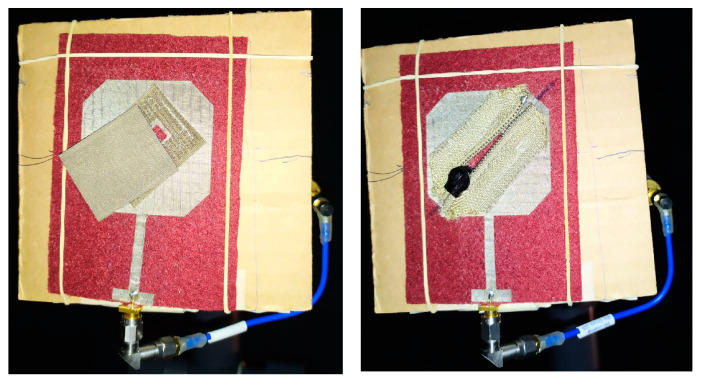
Repeatability measurements (4 of 10 use cases) of Velcro (**left**) and zip (**right**) clothing components.

Dedicated evaluation scenarios and measurement methodologies can be used for the experimental evaluation of WTA which applies to clothing components. In addition to the described scenarios, in the CC performance evaluation, further scenarios should be examined including the nature and use of the CC. In [48], the use of a conductive Velcro and a zip fastener in reconfiguring the polarization properties of a textile patch antenna was presented and examined in terms of repeatability. Specifically, the consistency of antenna performance after 10 cycles of using the CCs was examined. These 10 cycles can represent different users’ manipulation of the clothing components (Figure 13). These repeatability measurement scenarios take into account the mechanical nature and reconfigurable use of these CCs. A statistical analysis was then presented to prove an acceptable performance of these components. The Velcro tapes yielded a more stable and repeatable antenna performance than the zip. Still, the zip enabled an adequate antenna performance. Furthermore, in [40] the use of conductive snap-on buttons proved to present excellent repeatability characteristics based on measurements of the reflection S11 and transmission S21 parameters.

**Figure 14:**
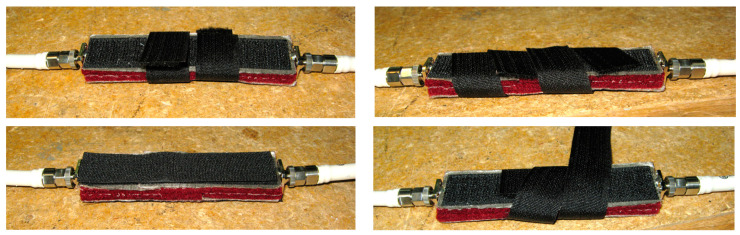
Non-conductive use of Velcro for interconnection of textile striplines in various ways of usage.

**Figure 15:**
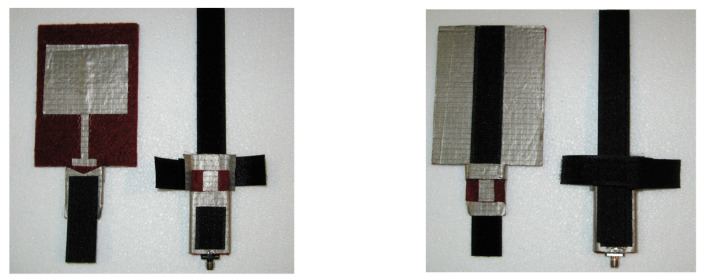

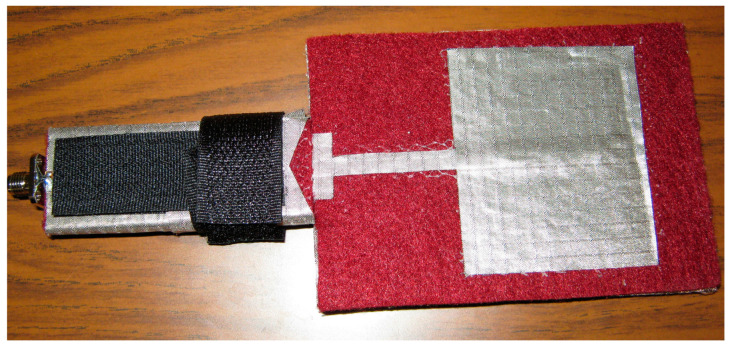
Non-conductive use of Velcro for interconnection of textile stripline with textile patch antenna.

There are cases that the clothing components can be used as a means of antenna feed attachment and interconnection as in [47]. In that specific application of a CC, it is useful and critical to experimentally examine the most effective way of using a non-conductive component in terms of performance. A non-conductive Velcro, as presented in [47], has been examined in terms of practical use and interconnection between textile transmission lines in [127]. Some concepts that were examined are shown in Figure 14. The results of transmission losses indicated that this textile fastener can be a proper candidate for the development of a practical “user-friendly” wearable system. Furthermore, the effective use of the non-conductive Velcro for attaching the feed transmission line of a textile patch antenna is shown in Figure 15. Measurements of the performance of the interconnection of the transmission line with the textile patch antenna have been carried out and validated the concept. Therefore, an all-textile antenna structure has been developed to enhance wearability by avoiding the use of rigid connectors.

As previously described, the zip has been used not only as a part, or a means of specific antenna geometry configuration but as an antenna itself. In the case of the zip monopole antenna [49,50], various lengths of the open zip state have been investigated. This analysis revealed the tunable property of a zip. Still, measurements should be carried out to assess the repeatability of the various tuning positions of the opened zip length. Furthermore, in the case of the handbag zipper antenna [51], various locations for positioning the feed probe of the SMA connector were examined to improve the antenna performance. It seems that the nature of the zip can offer the ability of different feed locations compared with a conventional textile antenna, e.g., monopole antenna. Therefore, testing different feed locations on a zipper antenna structure belongs to this specific category of CC experimental evaluation.

In [44], a modified snap-on button which includes a DC voltage PCB control circuit with a diode has been designed and examined, firstly, as a PCB in terms of circuit analysis and, then, embedded into the antenna structure for evaluating the whole system. It can be seen that the clothing components can be modified to include a PCB circuit. Furthermore, in [43], commercial metallic snap-on buttons have been used as detachable shorting vias for wearable textile antennas. The target is to achieve antenna modularity in terms of radiation characteristics. In the proposed design concept, four male snap-on buttons integrated into the substrate are soldered back to back to serve as antenna patch holders and as shorting vias when they are engaged to female buttons onto the ground plane. By selecting the engagement of different male buttons with the female ones, the textile antenna can be shorted at different corners and, thus, operate in different radiation modes.

In [18], realistic structural modifications of a button have been examined in terms of simulations and measurements. The implemented modifications can introduce a novel type of dual-band button antenna that enables wireless local area network applications and can be efficiently integrated into clothing. The proposed button design is suitable for on-body and off-body wireless communication. Similar experimental evaluations of a button antenna, in terms of realistic modifications and antenna locations with respect to the cloth, textile cover, and support substrate (integrated onto cloth) are presented in [28].

In [23], a dual-band button antenna has been designed following the structure of a typical jeans button for use in personal area networks (PANs). A point-to-point transmission measurement study has been carried out for various button locations. Possible positions are on the shoulders, in breast pockets, and in jacket buttons to represent realistic wearing conditions.

## 5. Potentials of Textiles Technology in Wearable Antennas

Textile technology is a well-established field where advanced techniques are employed to develop and manufacture various types of yarns and fabrics tailoring textile properties. Further, as has been outlined from the above analysis, the utilization of clothing components in wearable antenna technology has gained significant technological and scientific interest in the last years connecting the traditional sewing/embroidery industry with this futuristic e-textiles sector. The increasing demand for wireless functionalities within smart clothing could initiate new potentials of textile technology in the wearable antenna field. The potential could be general in terms of market and industry and specific regarding the clothing design and manufacturing procedure. The potentials can be listed as follows: (i) textile market expansion into electronics and antenna technology, (ii) manufacturing of clothing components to serve dual roles (clothes and antenna), (iii) low-profile and user-friendly antenna, and (iv) smart clothes for various applications taking into advantage (in terms of an antenna and electromagnetic performance) the existence of the mandatory clothing components. The following paragraphs expand each potential to offer a more crystal-clear view.

The textile market has been a great economical sector for decades as clothes are an indispensable daily industrial product. Still, clothes can be altered into novel high-tech products via the combination of wearable electronics and antenna devices. Wearable technology is among the advancing parts of entertainment, health, and safety. This combination will give a great boost to wearable technology and could enable chances for the mass production of smart clothes for various applications.

Presuming that a smart cloth is designed for a specific application (e.g., WLAN) by employing a clothing component as described in Section 2, then this component should be designed for a dual role: to be part of the cloth and part of the antenna, as well. Under these conditions, textile technology should upgrade the characteristics of the clothing components to allow for a more efficient exploitation of wearable antenna design. For instance, new buttons with satisfactory electrical conductivity could be developed, or zips in a closed state could create a more solid structure without gaps. Additionally, this (and in combination with the previous potential) will dramatically improve the specification of products in both technological sectors (textile and wearables).

As can be easily distinguished from Section 2, the wearable antennas when using the clothing components should be low-profile, user-friendly, somehow “hidden” in garments, and made from textile materials, which in contrast with rigid materials are appropriate and aesthetically acceptable by users.

Finally, all the above could enable from scratch design and production of smart clothes for various applications and communication systems (ISM, Bluetooth, WLAN, UWB, 5G, and IoT) which will efficiently exploit clothing components appropriate both for garment and wearable electronics functionalities.

## 6. Conclusions

In this paper, a complete review of commercially available clothing components applied within the framework of wearable textile antenna technology has been presented. CCs integrated into cloths can generate wireless communication functionalities that improve the user’s life quality via flexible and lightweight solutions.

Firstly, various types of CCs that can operate as radiating elements or as structural parts of a textile antenna were thoroughly investigated. It was outlined that low-profile and user-friendly clothing item designs can be suitable candidates in wearable antenna technology. Button shape designs have successfully acted as wearable antennas after their electrical characterization and their properties adjustment. The available metal structures, apart from the mechanical support, acted as the radiating parts of the wearable antennas. Furthermore, snap-on buttons were studied in terms of wearable antenna design and implementation. It was shown that modular design solutions can be developed to reconfigure antenna performance characteristics (e.g., resonance frequency, polarization, and radiation pattern) for multi-functional wearable systems. Furthermore, active components and biasing circuits can be embedded into compact configurations of snap-on buttons.

Velcro tapes have been, also, examined. The non-conductive Velcro can be used as a structural part of the textile antenna to allow interconnection with a feed line for instance, whereas, the conductive Velcro can be a functional part providing reconfiguration schemes. Regarding the zip fasteners, it was highlighted that the variety of their different open state positions can be exploited to offer reconfigurability in textile antenna designs. Each clothing component can be selected to be used as part or as an autonomous wearable textile antenna upon the desired application (e.g., ISM, WLAN, WiMAX, Bluetooth, UWB) and their electromagnetic performance.

Secondly, a five-step procedure for the design of textile antennas that incorporate CCs has been recorded, reviewed, and examined based on the authors’ studies and research works found in the literature. In the first step of Concept Selection, the desired application type, the applied wireless technology, the antenna operation, the type of antenna, and the usage of CC (as part of the antenna/or as the main radiator) are taken into consideration leading to a basic antenna design. Subsequently, the textile materials including conductive fabrics, dielectric fabrics, and CCs need to be electrically characterized. In the next step, a reference antenna model without employing a CC is designed and numerically simulated. Then, a prototype can be fabricated to evaluate the properties of the textile materials and the principle of operation of the reference antenna. In the fourth step, the clothing component is integrated into the reference antenna structure. Antenna design modifications are possible to cover the requirements set by the reference model. The last step is to validate the CC textile antenna model through measurements.

Furthermore, experimental evaluation scenarios of a CC textile antenna have been described. The scenarios include antenna measurements in free-space and onto the human body. Structural (e.g., bending) and environmental (e.g., humidity) conditions should be taken into account. Additionally, measurement technique considerations have been presented with respect to the experimental evaluation of significant antenna parameters including the reflection coefficient and radiation properties on a human phantom. Repeatability measurements seem to be necessary to obtain an adequate evaluation of the resonance behavior of textile antennae. In terms of the far-field radiation properties, the cylindrical near-field technique (CNF) has been found to be an effective measurement technique for on-body antenna measurements. Also, SAR needs to be numerically and experimentally evaluated in terms of practical applications and safety issues. Moreover, concerning the CC performance evaluation, the material, structure, and use of the clothing components need to be examined. Repeatability measurement scenarios that employ various users’ manipulation of the clothing component can be conducted taking into account the CC mechanical operation and reconfigurable use.

In general, the use of clothing components in the wearable textile antenna technology can combine the traditional sewing/embroidery industry with this scientific sector creating novel potentials such as textile market expansion into electronics and antenna technology, manufacturing of clothing components to serve dual role (clothes and antennas), low-profile and user-friendly antenna, and smart clothes for various wireless applications.

## Figures and Tables

**Table 1:** A performance comparison of button antenna designs found in the literature with respect to antenna type/radiation, dimensions, application (App.), frequency of operation (f_c_), 10-dB impedance bandwidth (BW), radiation efficiency (e_rad_) and far-field gain (Gain), where available.

Ref.	Type/Radiation	Dimensions (mm) L, W, H	App.	f_c_ (GHz)	BW (GHz)	e_rad_ (%)	Gain (dBi)
[11]	Top Monopole/ Omnidirectional	40, 40, 13.39	ISM/Wi-Fi	2.45	0.13	N/A	N/A
[13] *	Top Monopole/ Omnidirectional	17, 17, 15.35	ISM	4.5	0.65	N/A	2.2
[14]	Triangle patch-FSS/ Broadside	30, 30, 5	WLAN	5.25	0.6	70	5.1
[15]	Disc Monopole/ Omnidirectional	17, 17, 11	WLAN	2.4 5.2	0.147 1.404	N/A	2.4
[16]	Disc Monopole/ Omnidirectional	17, 17, 11	WLAN	2.4 5.35	0.147 1.404	N/A	2.4
[17]	Disc Monopole/ Omnidirectional	17, 17, 12.8	WLAN	2.4 5.25	0.159 1.605	N/A	0–3
[18]	Patch/ Broadside	16.02, 16.02, 4.575	ISM/WLAN	2.4 5.2 5.8	0.09 2.29	91.9 87.3 85.3	1.05 4.5 6.04
[19]	E-shaped PIFA patch/ Omni-Broadside	N/A	ISM	2.45 5.8	0.1 0.9	Ν/A	−0.8 6.8
[20]	Patch-Monopole/ Omni-Broadside	16.02, 16.02, 4.575	ISM	2.4 5.2 5.5	0.09 2.29	91.6 87 85	1.05 3.4 6.08
[21]	Disc Monopole/ Omnidirectional	17, 17, 8.9	WLAN	2.45 525	0.294 1.82	N/A	2.2 3.6
[22]	G-shaped Monοpole/ Omnidirectional	Complex Shape	ISM	2.45 5.8	0.08 0.35	N/A	N/A
[23]	Disc Monopole/ Omnidirectional	16 (50 GND), 16 (50 GND), 13.1	WLAN/ HiperLAN/2	2.45/5.2/5.5	0.1 1.44	N/A	N/A
[24]	Disc Monopole/ Omnidirectional	14.6 (60 GND), 14.6 (60 GND), 12.1	WLAN	2.4 5.5	0.165 1.15	89 85	2.65 5.1
[25]	Disc Monopole/ Omnidirectional	14.5, 14.5, 11.5	Bluetooth/ WLAND	2.45 5.25/5.5	0.3 2.3	N/A	N/A
[26]	Top monopole spiral-like/ Omni-Broadside	Complex details inside multi-parametric	ISM/WLAN	2.45 5.8	0.07 0.3	46.3 69.3	−0.6 4.3
[27]	Top monopole- Crossed-dipole/ Omni-Broadside	18, 18, 10	ISM/WBAN	2.45 5.8	0.18 3.38	N/A	2.2 8.6
[28]	Fractal/ Broadside	19.5, 19.5, 18.5	U-NII-5G/ WLAN	4.55 5.2	0.11 0.2	85.5 92.5	7.45 7.5
[29]	Top Monopole/ Omnidirectional	24 (60 GND), 24 (60 GND),18.2	UWB	3.05–10.95 (4/6.5/9)	7.9	Ν/A	3.45/5.1/5.6
[30]	Circular Monοpole/ Omnidirectional	15 (20.8 GND), 15 (36 GND), 9	UWB	3.1–10.6 (3.5/6/9)	>7.5	N/A	2.6/1.9/ 3.4
[31]	Top Conical Monopole/ Omnidirectional	26, 26, 12.5	UWB	3–12 (3.5/6/9)	9	N/A	3.9/2.8/4
[32]	Crossed-dipole- Annular ring/ Omni-Broadside	41.4, 41.4, 15	UWB ISM/WLAN/5G	4.12–9.12 2.45/3.7/5.85	5 0.16/0.35/0.39	>90 40–70	6–7.8 1/6.2/4.5
[33]	Hexagonal Patch/ Broadside	10, 8.66, N/A	WiGig/WBAN/Medical	62.12	11.91	N/A	5
[34]	Button Array (Disc Monopole)/Directive	10, 10, 4.8 (Single element)	WiGig	59	1.1	N/A	35
[35]	Planar Disc Monopole Multi-Ring/ Multidirectional	49.7, 31, 0.508 (substrate)	WiGig	61	4.27	65	4.7
[36]	Patch/ Broadside	33, 33, 5	ISM	0.868	0.25	1.5	−11.8
[37]	Top-monopole-loop loaded/Broadside	19.54 (100 GND), 19.5 (100 GND), 4.12	U-NII	5.5	0.68	N/A	3.8
[38]	Magnetic dipole-patch loaded/ Omnidirectional	19, 19, 10.29	WBAN	5.8	0.23	72.6	2.1
[39]	Ring/Various	15, 15, 10.087	ISM/5G	2.45	0.658	97	1.8

* Other buttons are tested, and other frequencies resonate at them.

**Table 2:** Performance comparison of snap-on button antenna designs found in the literature with respect to antenna type/radiation, dimensions, application (App.), frequency of operation (f_c_), 10-dB impedance bandwidth (BW), radiation efficiency (e_rad_), and far-field gain (Gain), where available.

Ref.	Type/ Radiation	Dimensions (mm) L, W, H	App.	f_c_ (GHz)	BW (GHz)	e_rad_ (%)	Gain (dBi)
[40]	Patch/ Broadside	15.2, 7.5, 3.5 (40 × 40 GND)	ISM, X-band	8	0.6	N/A	9.2
		13.4, 7.5, 3.5 (40 × 40 GND)	ISM, X-band	9	0.8		9.3
		11.8, 7.5, 3.5 (40 × 40 GND)	ISM, X-band	10	1.4		9.2
[41]	Various Patches/ Omni-Broadside	Complex details	ISM, WLAN	5 (RHCP)	0.55	N/A	5
			ISM, WLAN	5 (LHCP)	0.5		5
			ISM, WLAN	2.45 (LP-PIFA)	0.1		3.5
			ISM, WLAN	5.3 (LP)	0.3		7.8
			ISM, WLAN	4.72/5.1/5.26 (LP-SLOT)	0.25–0.8		N/A
			ISM, X-band	8 (FOLDABLE)	0.5		8.9
[42]	Circular Patch/ Broadside	23.4, 23.4, 3.5 (40 × 40 GND)	ISM/WLAN	5 (CP) 4.7 (Slot) 5.2 (No-Slot)	0.5 0.6345 0.494	N/A	N/A
[43]	Patch/ Broadside	18.5, 19.5, 3.5 (40 × 40 GND)	ISM	3.62	0.1	N/A	N/A
[44]	PIFA or Patch/ Broadside	23, 20, 3.2 (60 × 55 GND)	ISM/WLAN	2.45 5.8	0.093 0.234	30–90.2 80.4–88.7	−0.9–5.9 8.1–8.4
[45]	PIFA/ Broadside	26, 22, 3.2 (60 × 50 GND)	ISM	1.82–2.94	0.071–0.153	29.8–93.7	−1.07–7.37
[46]	CMSIC/ Omni-broadside	120, 79.7, 3.2 (120 × 120 GND)	ISM	2.45	N/A	OFF:82.7 ON:77.1	OFF:9.2 ON:3.8

**Table 3:** Performance comparison of Velcro patch antenna designs found in the literature with respect to antenna type/radiation, dimensions, application (App.), frequency of operation (f_c_), 10-dB impedance bandwidth (BW), radiation efficiency (e_rad_), and far-field gain (Gain), where available.

Ref.	Type/ Radiation	Dimensions (mm) L, W, H	App.	f_c_ (GHz)	BW (GHz)	e_rad_ (%)	Gain (dBi)
[47]	Patch/ Broadside	49, 57.6, 4.26 (100 × 70 GND)	ISM/WLAN	2.486	0.09	85.76	7.80
[48]	Patch/ Broadside	53, 53, 4.2 (100.4 × 74.2 GND)	ISM/WLAN	2.47 (LP) 2.35 (CP)	0.103 0.170	77.1 79.5	6.10 6.88

**Table 4:** Performance comparison of zip antenna designs found in the literature with respect to antenna type/radiation, dimensions, application (App.), frequency of operation (f_c_), 10-dB impedance bandwidth (BW), radiation efficiency (e_rad_), and far-field gain (Gain), where available.

Refs.	Type/ Radiation	Dimensions (mm) L, W, H	App.	f_c_ (GHz)	BW (GHz)	e_rad_ (%)	Gain (dBi)
[48]	Patch/ Broadside	53, 53, 4.2 (100.4 × 74.2 GND)	ISM/WLAN	2.39 (LP) 2.31 (CP)	0.113 0.142	62.3 60.2	4.97 5.67
[49,50]	Zip monopole/ Omnidirectional	57, 6, 2 (30 × 40 GND)	ISM/WLAN/ Bluetooth	2.5 5.6	0.95 N/A	N/A	0
[51]	Zip monopole/ Various	385, 13, 21.535	ISM/WLAN/ Bluetooth/IoT	2.2 2.44 2.53	0.12	98.4 99 98.4	4.72 4.74 5.79
[52]	Zip monopole/ Various	Complex	ISM/WLAN/IoT	2.4 5.5	N/A	N/A	7.01 7.68

**Table 5:** Results of dielectric properties measurements of three felt samples of thickness 2.64 mm along directions A and B: dielectric constant (ε_r_), and loss tangent (tanδ) @2.471 GHz.

Direction	Samples	ε_r_	tan δ
A	A1	1.404	0.0198
	A2	1.359	0.0184
	A3	1.335	0.0173
B	B1	1.396	0.0187
	B2	1.377	0.0186
	B3	1.376	0.0187

**Table 6:** Simulated and measured performance results for reference antenna frequency of operation (f_c_), reflection coefficient at f_c_ (S11), 10-dB impedance bandwidth (BW), the axial ratio at f_c_ (AR), radiation efficiency (e_rad_), and far-field gain (Gain).

Antenna		f_c_ (GHz)	S11 (dB)	BW (MHz)	AR (dB)	e_rad_ (%)	Gain (dBi)
REF1	Simulations	2.45	−22	70	55	96	7.78
	Measurements	2.47	−35	79	18	94.7	6.90
REF2	Simulations	2.33	−9.1	120	1.9	94.7	8.20
	Measurements	2.34	−10	160	3.1	94.7	7.44

**Table 7:** Simulated performance results for reference antenna (Pins) and CC antenna (Snap-on buttons): frequency of operation (f_c_), reflection coefficient at f_c_ (S11), 10-dB impedance bandwidth (BW), radiation efficiency (e_rad_), and far-field gain (Gain).

Antenna	f_c_ (GHz)	S11 (dB)	BW (MHz)	e_rad_ (%)	Gain (dBi)
REF (Pins)	2.45	−29.6	83.2	69.9	1.2
CC (Snap-on buttons)	2.446	−29.5	83.6	69.1	1.1

## Data Availability

We do not have online research data base.
